# Valorization of Recycled Gypsum from CDW in Green Binder Systems

**DOI:** 10.3390/ma18214849

**Published:** 2025-10-23

**Authors:** Roumiana Zaharieva, Borislav Simonov

**Affiliations:** Centre of Competencies “Clean&Circle”, University of Architecture, Civil Engineering and Geodesy, 1 Hristo Smirnenski Blvd., 1046 Sofia, Bulgaria; simonov_fce@uacg.bg

**Keywords:** recycled gypsum, natural zeolite, recycled brick powder, green binder, compressive strength, softening coefficient, XRD, DTA/TG

## Abstract

The circular economy in construction requires the valorization of gypsum waste from construction and demolition. Waste from gypsum plasterboards is considerable, yet it is still viewed more as a problem than as a mineral resource. This study investigates the potential for utilizing recycled gypsum (RG) from waste plasterboards in the production of blended green binders. Four gypsum–cement–pozzolanic binders are designed with two pozzolanic additives (natural zeolite and recycled brick powder) in two ratios to cement—0.6 and 1.0. The structural mineral compounds of the binders are analyzed by XRD and DTA/TG, while the performance of both fresh and hardened paste is evaluated by standardized methods for binders to determine possible construction applications of these green binders. Results show that RG can be used to produce blended fast-setting binders with a gypsum content of above 40%. Systems with natural zeolite achieve higher strength (up to 30 MPa at 90 days) and sufficient water resistance, thus suitable even as substitutes for cement binders. The developed blended binders with recycled brick powder can be used in low-moisture environments only as substitutes for gypsum binders in plasters, masonry units, and lightweight composites.

## 1. Introduction

Global cement production is expected to increase by up to 23% by 2050 [1]. Despite significant progress over the past 10–15 years in reducing greenhouse gas emissions from cement production through the use of alternative fuels [2,3], alternative raw materials [4], mineral additives, including those derived from industrial waste [5], as well as emission capture [6,7], Portland cement clinker remains the primary source of emissions in the construction sector [8]. Consequently, efforts are focused on developing alternative binders with a lower environmental footprint, possessing construction and technical properties that allow the replacement of cement in certain applications. Examples of such alternative binders include geopolymers [9,10], binary [11,12,13], and ternary blended binders [14,15,16], special cements such as calcium aluminate and calcium sulphoaluminate cements [17,18].

At the same time, the concept of a circular economy in construction [19,20] and the pursuit of more sustainable resource utilization [21,22,23] require that construction and demolition waste (CDW) be valorized to the maximum extent. According to [24], the construction industry consumes over 55% of natural resources and generates approximately 30% of the total waste worldwide [25]. Many mineral CDWs, such as concrete and bricks [26], as well as metals [27] and asphalt concrete [28], are widely recycled, but other waste streams are considered problematic from a treatment perspective. Such are gypsum-containing wastes, which amount to around 15 million tons per year globally [29]. In the amendment to the Waste Framework Directive in 2018 [21], the gypsum wastes were included in the target groups for separate collection and recovery [30]. They consist of production waste (pre-consumer waste) and CDW (post-consumer waste), originating from masonry units, boards, panels, decorative elements, and gypsum plasters, with the largest share being gypsum plasterboards generated during renovation, reconstruction, and building demolition [31,32]. Given the increasing volume of dry construction systems in developed countries, gypsum plasterboard waste is expected to continue growing [33,34].

Gypsum is relatively easy to recycle from a technological standpoint, and the processes have a low environmental footprint [32,35]. However, due to the diversity of gypsum-containing wastes and the comparatively small quantities generated at individual sites, which increases logistics costs, the valorization of post-consumer gypsum-containing CDW remains somewhat limited at present [34,36,37]. Successful projects have also been carried out using pre-consumer gypsum waste [34].

Gypsum-containing CDWs require special handling during disposal, as well. They are not inert and therefore cannot be disposed of in inert waste landfills. On the other hand, they cannot be disposed of together with biodegradable waste, as this leads to the formation of toxic hydrogen sulfide [38] and contributes to methane (CH_4_) generation [39]. Such wastes must be collected separately and either stored on different sites or disposed of in special cells within landfills [40,41].

Consequently, recycling gypsum-containing CDW would reduce both landfill pressure and pollution from this type of CDW, thus turning it into a valuable material that can be reused in the construction industry.

Gypsum is among the oldest known construction materials [42,43], yet it remains one of the most widely used in modern construction [44,45]. Gypsum binders are applied in the production of gypsum pastes (plasters and mortars) and gypsum products (blocks, panels, boards, decorative elements), since they are characterized by good workability, rapid setting and hardening, satisfactory mechanical properties, thermal and acoustic performance, and increased resistance to high temperatures. A major drawback of construction gypsum (calcium sulfate hemihydrate), including the recycled one, is its low water resistance [46,47], which classifies it as an air binder and constrains to some extent the application of gypsum products.

The present study aims to determine the potential for using recycled gypsum (RG) obtained from waste gypsum plasterboards in the production of blended green binders with improved properties (higher strength and water resistance), compared to gypsum binders, and to outline appropriate fields of its application.

## 2. Blended Green Binder Systems Based on Gypsum

Blended green binder systems are used as an alternative to conventional binders in the production of masonry units [48,49], lightweight composites [50], plasters and mortars suitable for the restoration of historic buildings [14].

Among green binder systems, gypsum–cement–pozzolan binders (GCPB) stand out [15]. GCPB contains gypsum (G), pozzolanic additives (PAs), and cement (up to 40%), which serves as an alkaline activator [51,52,53]. In some cases, lime can be used instead of Portland cement [14,54,55]. GCPB provides several specific advantages, including rapid setting and hardening, and, unlike conventional gypsum binders, they exhibit higher strength and improved water resistance [15,51], allowing them, in some cases, to replace not only gypsum but also cement binders.

A gypsum–cement system with a gypsum content of above 40% would not be stable without the use of a pozzolanic additive, as it would initially harden rapidly, but after a few months, the formation of ettringite (the trisulfate form of calcium hydrosulfoaluminate) would cause deformations that would demolish it. Pozzolanic additives, with their active silicon dioxide, reduce the concentration of calcium hydroxide (CH) in the liquid phase, thereby creating conditions for the stability of the gypsum–cement–pozzolan system [51,56,57].

Furthermore, pozzolanic additives are widely employed in the production of CEM II B–P, Q, V, and CEM IV blended cement types. According to EN 197-1 [58], materials used as PAs for cements must contain more than 25% reactive silicon dioxide. PAs themselves have weak or no binding properties, but when sufficiently fine and in the presence of water and calcium hydroxide, they react chemically and thus cementing compounds (calcium hydrosilicates C-S-H) are formed [59,60]. GCPB can use as PA not only highly active industrial by-products (such as silica fume [52,53,61] and fly ash [15,48,49,50,55,62]), but also low-activity wastes (e.g., finely ground quartz, sandstone powder, etc.) [63,64]. Due to a very small particle size and high pozzolanic activity, waste from geothermal wells has been used as PA, according to [52,65]. Some mining wastes, such as ferruginous quartzite [51], can also serve as PA. Natural PAs include volcanic rocks such as zeolites, volcanic tuffs, various types of clays, etc. However, no data have been identified on the use of natural zeolite in gypsum-based water-resistant ternary binders. Clays often require additional thermal (i.e., energy-intensive) activation, with metakaolin being the most used PA derived from calcined clays [49,52,54].

Finely ground clay brick and roof tile powder (hereafter referred to as brick powder, BP) may also exhibit pozzolanic activity [66,67] and can be used as PA even in blended cements [68,69]. However, their pozzolanic activity varies widely (from very low to high) depending on the mineral composition of the clay and the firing conditions of the bricks and tiles [70]. Research on the application of BP in ternary systems remains limited to low-strength binders based on lime and gypsum [14].

With regard to CDW gypsum (mainly from plasterboards), studies confirm its good recyclability for the production of plasterboards [34,71], but no applications of recycled CDW gypsum for the production of ternary gypsum-based water-resistant binders, achieving compressive strength up to 30 MPa, have been reported.

## 3. Materials and Methods

### 3.1. Materials

#### 3.1.1. Recycled Gypsum (RG)

Gypsum is a hemihydrate obtained from the recycling of CDW plasterboards of type “A”, manufactured by Knauf Bulgaria EOOD (Sofia, Bulgaria) in accordance with EN 520 [72]. The plasterboards are produced using synthetic FGD gypsum from the “Maritsa East 3” Thermal Power Plant, situated near the village of Mednicarovo in Bulgaria.

**Figure 1:**
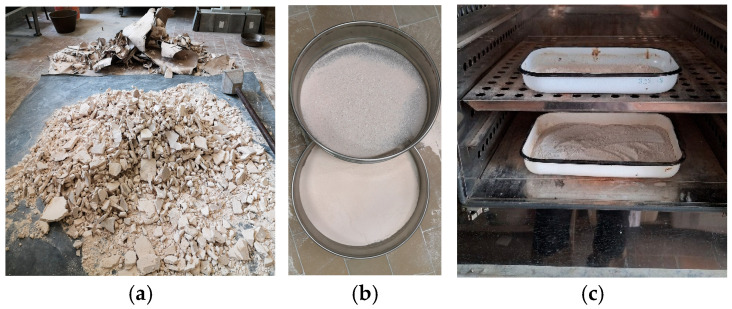
Recycling of CDW gypsum in laboratory: (**a**) Fragmentation; (**b**) Sieving; (**c**) Calcination of fraction 0/0.25 mm.

Plasterboard CDWs were recycled under laboratory conditions. The recycling process included crushing, manual removal of the cardboard, and additional fragmentation of the gypsum core (Figure 1a). The gypsum waste was then ground in a ball mill and sieved through a 0.25 mm mesh; thus, coarse particles of gypsum and paper were removed (Figure 1b). The fraction used for GCPB was 0/0.25 mm and was spread in about 2–3 cm-thick layers in trays. After that, analogous to the recycling technology applied in [73,74], it was heated in a ventilated laboratory oven at a temperature of 140 °C for 4 h (Figure 1c). The calcined gypsum was cooled in a desiccator and subsequently stored in sealed polyethylene bags to prevent interaction with the humidity in the air.

**Figure 2:**
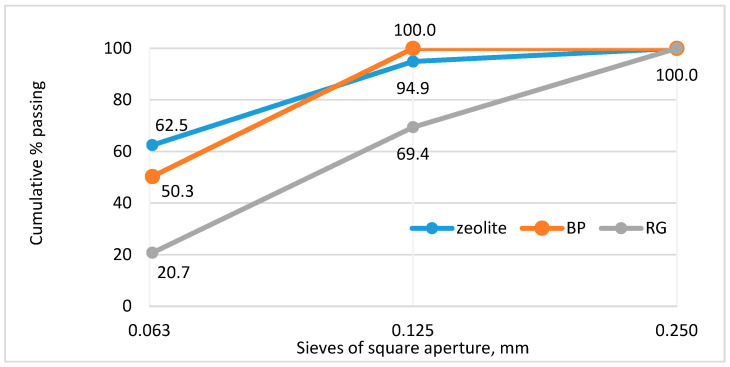
Grading of mineral compounds used.

The grading of recycled gypsum as per BDS EN 933-1 [75] is shown in Figure 2.

**Figure 3:**
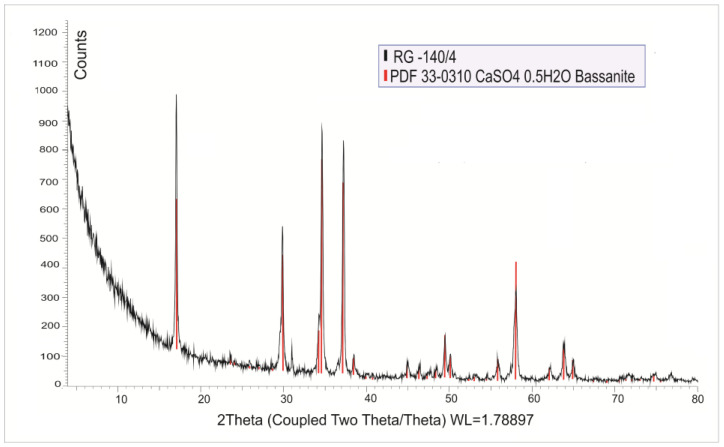
XRD of the recycled gypsum.

The mineral composition of the recycled gypsum was determined by XRD analysis (Section 3.4.1). It was found that the crystalline phase of the recycled gypsum consisted entirely of bassanite (Figure 3). No traces of dihydrate gypsum or anhydrite were present.

RG properties were determined primarily by standardized methods (Section 3.4) and compared with those of a sample of conventional construction gypsum (CCG), defined according to EN 13279-1 [76] as gypsum binder type “A” (Table 1). Natural gypsum was used as the raw material for the production of CCG.

RG is characterized by a lower bulk density (by approximately 20%) compared to CCG (Table 1), as likewise reported by other authors [81]. According to them, this is due to the different arrangement of the crystals—organized and compact in CCG, while dispersed and spaced, with large voids, in RG. The other reason for the lower bulk density of RG is that successive hydration and dehydration reduce the size of the molecules and increase the size of the voids.

The water demand of RG is 33% higher compared to that of CCG. A similar result has also been reported by [74]. The increased water demand might be due to the origin of the raw material—the FGD gypsum used for the plasterboards, and subsequently the RG itself, has finer crystals compared to the crystals of natural gypsum and CCG. In addition, the presence of microscopic paper particles in RG from plasterboards, observed by [81] using a scanning electron microscope (SEM), likely contributes to the higher water demand, as cellulose is hydrophilic in nature.

For completeness, although this is not required for CCG, to better characterize the specific features of RG, not only the initial but also the final setting time was determined, following the procedure of a now-withdrawn standard [79]. It has been established that both the initial and the final setting of the RG used occur much more rapidly at the 3rd and 8th minute, respectively, compared to CCG (Table 1). Comparison with other studies on the setting time of recycled gypsum is difficult, since different methods for determining setting times are often applied [74,81].

The conducted tests show that RG exhibits relatively high strengths, but they are lower than those of CCG, by about 17% in compressive strength and about 30% in flexural strength (Table 1). This is probably due to the fact that there might be interlocking of the crystalline structure and more defects in the gypsum crystals in RG, rather than well-formed crystals and an orderly structure as in CCG [81].

#### 3.1.2. Portland Cement

Ordinary Portland cement (OPC) CEM I 52.5R (HOLCIM Bulgaria AD, Beli Izvor, Bulgaria) was used, in accordance with EN 197-1 [58]. The physicomechanical properties of the cement were determined experimentally and are presented in Table 2.

#### 3.1.3. Pozzolanic Additives (PAs)

Two types of PAs were used: ground natural zeolite (Z) and recycled clay brick powder (BP). In accordance with BDS 166 [84], the pozzolanic activity of PAs was evaluated based on the amount of CaO [mg] that was bound by 1 g of PA after 30 days. As specified in BDS 16720 [85], PAs were classified according to their activity (Table 3).

Zeolites are crystalline three-dimensional microporous aluminosilicate minerals with pozzolanic properties, which is why they are also used in construction [86]. Natural zeolites are highly porous, hydrated aluminosilicates with a rigid crystalline structure and a network of interconnected tunnels and cages (such as a honeycomb) [87]. Many types of zeolites are known in nature, among which clinoptilolite is the most common and widely used, thanks to its unique structure, exceptional ion-exchange properties, and high reactive silica content [88].

The zeolite employed in this study is a natural material of Bulgarian origin, marketed under the trade name Vivolith 85 and produced by S&B Industrial Minerals SA (Kifissia-Athens, Greece). Its mineral composition, determined by XRD, is presented in Table 4 and contains over 40% of sodium clinoptilolite and over 50% of calcium heulandite (another mineral of the zeolitic family). After additional grinding, a fineness of 3960 cm^2^/g was achieved according to EN 196-6 [82], comparable to that of the cement used. The grading as per BDS EN 933-1 [75] is shown in Figure 2. The measured pozzolanic activity is 178.8 mg/g, which classifies this PA as a highly active pozzolanic additive, in agreement with the limits presented in Table 3.

The pozzolanic activity of brick dust depends mainly on the content of the amorphous phase, which contains possibly active SiO_2_, as well as on the reactivity of the minerals contained therein. Muscovite and microcline, in their natural state, are weakly active, but can exhibit pozzolanic activity upon appropriate mechanical activation (by destroying their crystal structure and increasing the specific surface area) [89,90]

For the preparation of BP for the needs of the present study, CDW from hollow blocks produced in Bulgaria in the 1970s was utilized. The blocks were manually cleaned of the binding mortar (i.e., traces of mortar may remain in the BP) and subsequently ground in a ball mill to a fineness of 3870 cm^2^/g following EN 196-6 [82]. The grading as per BDS EN 933-1 [75] is shown in Figure 2. The mineral composition, based on XRD, is presented in Table 5. The potentially active pozzolanic compounds are the amorphous phase of 27%, microcline of 21% and muscovite of 9%. The measured pozzolanic activity as per BDS 166 [84] is 116.9 mg/g, which classifies this PA as a highly active pozzolanic additive, in agreement with the limits presented in Table 3.

#### 3.1.4. Chemical Admixture

To improve the workability of the mixes, the Sika^®^ ViscoCrete^®^ GL 3070 (SIKA Bulgaria EOOD, Sofia, Bulgaria) superplasticizer was used.

### 3.2. Mix Design

#### 3.2.1. Composition Ratio of the Binder Components

In blended three-component binder systems, the gypsum content varies widely (20–80%) [14,15,51]. Since the present study focuses on the valorization of recycled gypsum, the amount of RG in the gypsum–cement–pozzolan binders (GCPBs) was set at 45%. A similar content has also been used in the studies of [14,15,51,52].

The amount of pozzolanic additive was determined so that the GCPB system remains stable and does not undergo deformations due to ettringite formation. The methodology described in the Russian technical specifications TU 21-31-62-89 [91] was applied. It has also been used in the studies of [47,51,92]. Following this methodology, since the stability of the system depends on the concentration of calcium oxide (CaO) in the liquid phase of the binders, the PA:OPC ratio in a water suspension of hemihydrate gypsum, OPC, and PA was determined such that, for a given gypsum content, the CaO concentration on the 5th and 7th day would not exceed 1.1 g/L and 0.85 g/L, respectively. To this end, a water suspension was prepared with a constant G:OPC ratio of 1.6, while the PA:OPC ratio varied. For PAs with an activity above 200 mg/g, it is recommended that this ratio be set to 0.5, 1.0, and 1.5. Two identical series were prepared. The first series was tested on the 5th day, and the second on the 7th day. The CaO content in the filtered solution at the respective age was determined by titration with 0.1 N hydrochloric acid (HCl) solution in the presence of phenolphthalein. The formula for calculating the concentration of calcium oxide (CaO, g/L) is as follows:

(1) $$CaO=\frac{768.A.T}{B},$$

where

A—the amount of hydrochloric acid used for titration, ml;
T—the titer of hydrochloric acid (HCl content in g/mL);
B—the amount of binder subjected to titration, mL.

**Figure 4:**
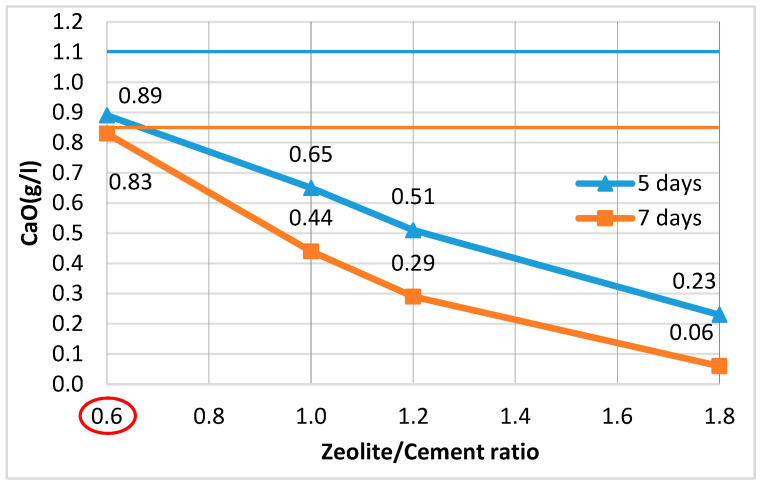
Variation in CaO concentration at different Z:OPC ratios.

**Figure 5:**
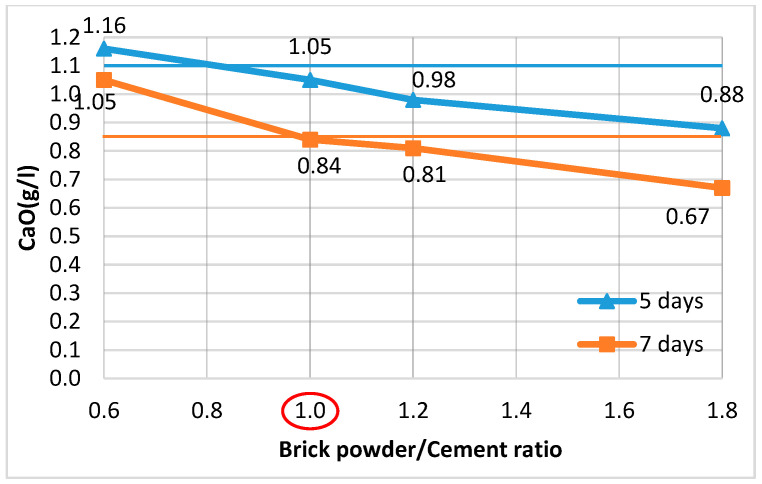
Variation in CaO concentration at different BP:OPC ratios.

The results obtained are used to construct graphs showing the change in CaO concentration on the 5th and 7th day for different PA:OPC ratios. The graphs allow the identification of the PA:OPC ratio at which the CaO concentration does not exceed 1.1 g/L on the 5th day and 0.85 g/L on the 7th day at the same time. Since the PAs considered in this study have lower activity than the additives used in the described methodology, the PA:OPC ratios were correspondingly increased to 0.6, 1.2, and 1.8, while the G:OPC ratio was kept constant at 1.6. Some authors, using a different approach to mix design and different PAs, recommend an optimal PA:OPC ratio of 1.0 [15,62], which is why it was selected as intermediate and the graphs were plotted using four points instead of three, as in the original methodology (Figure 4 and Figure 5).

It has been found that when using zeolite, it sufficed to be 60% of the cement mass (i.e., a Z:OPC ratio of 0.6) (Figure 4), whereas for brick powder, the system was stable at a higher BP content (OPC:BP ratio of 1.0) (Figure 5).

To ensure comparability of the results for the two PAs, further mixes were prepared using both PA:OPC ratios, namely 0.6 and 1.0.

The compositions of the mixes of the binders studied are presented in Table 6.

#### 3.2.2. Water-to-Binder Ratio and Plasticizing Admixture Amount

Based on preliminary tests on the workability of the mixes, performed according to a modified procedure of EN 1015-3 [93] (see Section 3.4.2), the water-to-binder ratio was chosen to be 0.7 (Table 6). Typically, when conventional gypsum and plasticizing admixtures are used, the water-to-binder ratio is lower [49,51,53]. However, as mentioned above, RG is characterized by an increased water demand (see Section 3.1.1).

**Figure 6:**
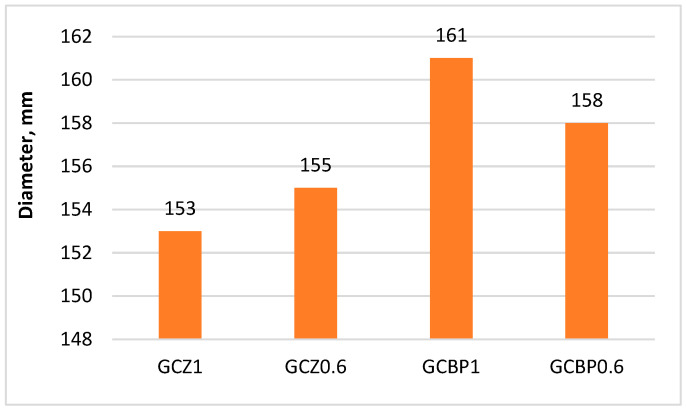
Consistency of fresh pastes.

The amount of admixture was determined so that the desired workability of the different mixes was in the range of 150–160 mm (Figure 6).

The plasticizing admixture is present in larger quantities in the mixes with a higher PA content (PA:OPC = 1.0), as they incorporate less RG and OPC, whose rheological behavior is influenced by the admixture. Due to the high hydrophilicity of zeolite, in pastes where zeolite is used (GCZ1 and GCZ0.6), the amount of plasticizing admixture exceeds that in pastes with BP (GCBP1 and GCBP0.6) (Table 6).

### 3.3. Samples Preparation and Treatment

For each mix presented in Table 6, twelve prisms measuring 4 × 4 × 16 cm were prepared. The molds were covered with polyethylene foil and stored for 24 h in a standard curing cabinet at a temperature of 20 ± 2 °C and RH of 95 ± 3%. Demolding was carried out at the age of 1 day.

Each prism was split in half after demolding, resulting in a total of 24 specimens from each mix, with 12 cured under one set of conditions and the other 12 under another. Two curing regimes were selected:

- the first, typically applied to gypsum binders, referred to as “air curing,” at a temperature of 23 ± 2 °C and RH of 50 ± 5%;
- the second, typically applied to Portland cement, referred to as “water curing”, in which the specimens are stored under water at a temperature of 20 ± 1 °C. The samples cured in water are designated with an additional letter W (e.g., GCZ1-W).

The results are presented as the arithmetic mean values obtained from the testing of three specimens.

The specimens for XRD and DTA/TG analyses (Section 3.4.1 and Section 3.4.4) were prepared as follows: after the compressive strength test, the specimens (approximately 4 × 4 × 8 cm) were subjected to partial drying in a ventilated oven at +60 °C for 4 h. From the central part of the specimens, a sample of about 1 cm^3^ was taken and manually ground to a particle size not exceeding 0.25 mm.

### 3.4. Methods

#### 3.4.1. XRD Analysis

To determine the mineral composition of gypsum, zeolite, and brick powder, an X-ray diffraction (XRD) analysis was carried out. The analysis was performed at the Faculty of Geology and Geography, Sofia University, using Powder X-ray Diffractometer BrukerD8 Advance (Bruker GmbH, Manheim, Germany), with a LynxEye detector (Bruker) and with Co Kα radiation, vertical θ/θ goniometer, and a step size of 0.02 (2θ). Bruker AXS DIFFRAC.EVA version 5.2.0.5 employing ICDD-PDF2 crystallographic database was used for the structural data and semi-quantitative phase analysis [94].

XRD analysis was also performed on the blended binders at the ages of 7, 28, and 90 days. In this case, given the accuracy of about 2% in the identification of crystalline phases, minerals with similar XRD profiles, such as tobermorite and hillebrandite, were grouped together in the presentation of the results. Quartz and albite, characteristic of BP, are considered inert, which is why they were likewise presented together. It is also possible that the peaks of the trisulfate and the lower/monosulfate forms of calcium sulfoaluminates exhibit a similar pattern [95].

#### 3.4.2. Determination of Properties of Fresh Pastes

Given the need to compare the characteristics of RG with those of conventional construction gypsum, the EN 13279-2 [78] standard was applied to determine the setting time and water/plaster ratio.

Bulk density is not a characteristic, which is usually declared for gypsum binders and that is why a methodology of EN 459-2 [77], applicable for building lime and other fine grained materials was used.

Density of hardened paste is determined according to EN 12390-7 [80].

Taking into account that the new binders must be water resistant to replace OPC in some applications, some methods used for cement testing were also used.

The consistence of the fresh GCPB paste was determined in accordance with EN 1015-3 [93] by the flow table method but using a modified procedure to account for the rapid setting of GCPB –shorter mixing time (only 25 s) and application of tapping 60 s after mixing water with the dry components (Figure 6).

The setting times (initial setting time and end of setting) of GCPB were determined using a Vicat apparatus (Utest Material Testing Equipment, Ankara, Turkey), in accordance with EN 196-3 [96].

#### 3.4.3. Determination of Properties of Hardened Pastes

The compressive strength of both recycled and conventional gypsum were determined according to EN 13279-2 [78].

Taking into account that the new binders must be water resistant to replace OPC in some applications, the strength of blended binder pastes was determined according to cement testing method, i.e., EN 196-1 [83] at the ages of 2, 7, 28, and 90 days. Before each compressive strength test, the specimens were inspected for cracks or other defects caused by ettringite.

The softening coefficient was determined to assess the water resistance of the GCPBs. It is usually defined as the ratio of the compressive strength of a water-saturated specimen to that of a dry specimen, with mixes considered water-resistant if the coefficient exceeds 0.8 [51,57]. Its determination normally involves drying the specimens at 105 ± 5 °C followed by re-saturation. Due to the risk of transformation of newly formed mineral phases [14] and cracking of the paste, in this study the coefficient was calculated as the ratio of the strength of a specimen cured under water (see Section 3.3) to that of a specimen cured under air curing conditions (see Section 3.3), according to Formula (2). The threshold of 0.8 was again adopted to classify the pastes as water-resistant.

(2) $$K_{sc}=\frac{R_{wet}}{R_{dry}},$$

where

$R_{dry\;}$—compressive strength of specimens after air curing;
$R_{wet}$—compressive strength of specimens after water curing.

#### 3.4.4. Combined Differential Thermal Analysis and Thermogravimetry (DTA/TG)

To monitor the type of new formations in the blended binders cured under different conditions (water- and air-cured), a total of 24 specimens at the ages of 7, 28, and 90 days were subjected to differential thermal and thermogravimetric analyses (DTA/TG). The analyses were carried out at the Institute of General and Inorganic Chemistry, Bulgarian Academy of Sciences. The measurements were performed using a Labsys Evo 1600 apparatus (Setaram, Caluire, France). The specimens were thermally treated over a temperature range from room temperature to 1000 °C. The heating rate was 10 °C/min in an air flow with baseline correction for DTA and thermogravimetric analysis using an empty reference sample.

## 4. Results and Discussion

### 4.1. Setting Time of GCPBs

**Figure 7:**
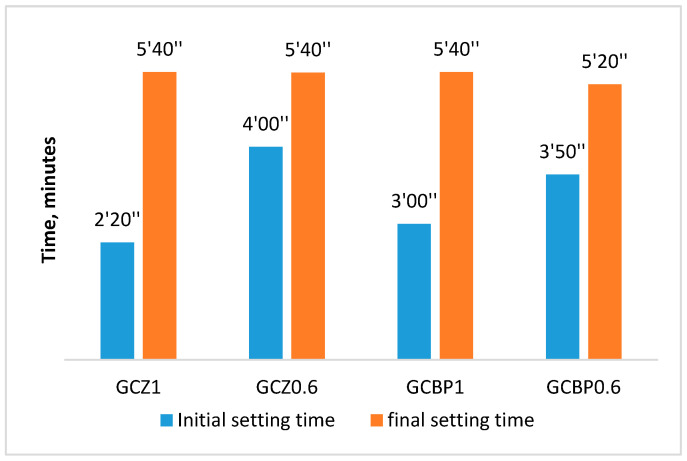
Setting the time of the different binders.

It has been found that the investigated GCPBs exhibit much faster initial and final setting times compared to both Portland cement and ordinary construction gypsum. The initial setting for all pastes occurs in less than 4 min. It is the shortest (under 3 min) for the pastes with lower cement content and higher PA content (GCZ1 and GCBP1). The end of setting occurs in less than 6 min (Figure 7).

It is noteworthy that for all pastes, the time from initial to final setting is very short (from about 1′30″ to about 3′30″), indicating that the produced GCPBs are extremely fast-setting. Other researchers have also reported the fast-setting nature of GCPBs [14,51,97]. Depending on the intended application, chemical admixtures (e.g., setting retarders) can be used to regulate the setting time of GCPBs [15,62].

### 4.2. Compressive Strength Kinetics

#### 4.2.1. Hardening Under Air Curing

**Figure 8:**
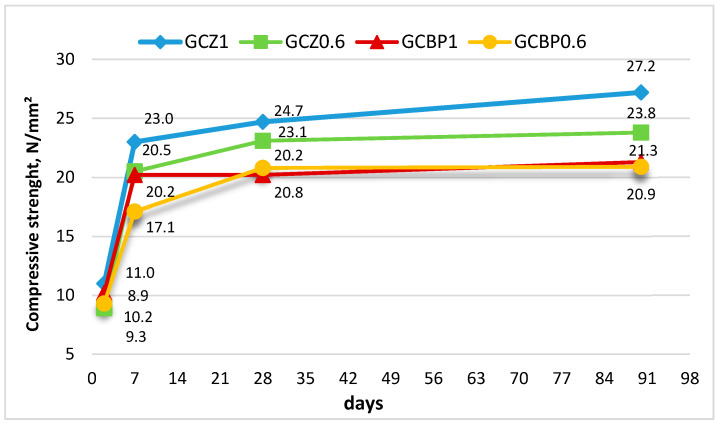
Compressive strength of specimens after air curing.

The rate of strength development under air curing is most pronounced at early ages, which can be attributed to gypsum hardening. On the 2nd day, the strengths of all pastes are about 10 MPa, increasing to approximately 20 MPa by the 7th day (Figure 8).

From the 7th to the 90th day, strength grows more gradually, driven by cement hydration, ettringite formation, and carbonation. The strengths of the zeolite-containing pastes reach about 24 MPa at the age of 28 days. At 90 days, the highest strength is observed for GCZ1 (27.2 MPa). The strengths of the BP-containing pastes (GCBP1 and GCBP0.6) reach approximately 20 MPa at 28 days and do not increase significantly between the 28th and 90th day, remaining about 22% lower than those containing zeolite (Figure 8).

#### 4.2.2. Hardening Under Water Curing

**Figure 9:**
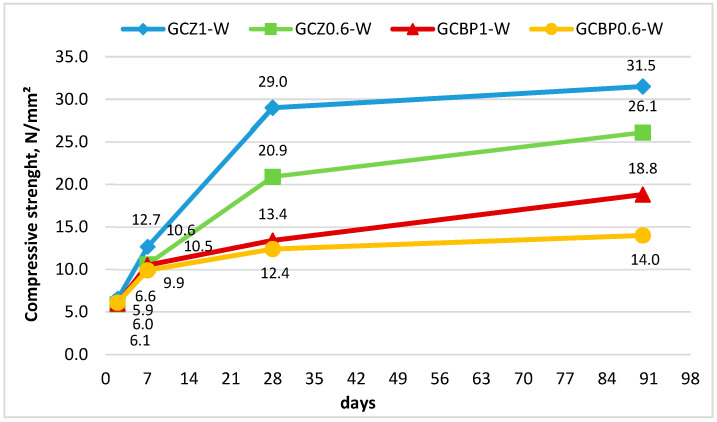
Compressive strength of specimens after water curing.

It has been found that hardening processes under water curing differ significantly from those under air curing. Early-age strengths under water curing are considerably lower: about 6 MPa on the 2nd day and around 10 MPa on the 7th day (Figure 9).

The reason for this lower early strength is the partial dissolution of gypsum, as by the 7th day, there is relatively little time for the formation of sufficient calcium silicate hydrates (C-S-H) to protect it from water [15,62].

Despite a substantial increase in compressive strength over the following weeks, 28-day strengths under water curing remain lower than those achieved under air curing, except for the paste with a higher zeolite content (GCZ1-W), where the measured strength is 29.0 MPa compared to 25 MPa for the same paste under air curing (GCZ1). The results are consistent with those reported in [62], where similar pastes using fly ash as PA (G:OPC:PA = 40:30:30) achieved comparable performance.

Water curing favors the pozzolanic reaction, and for pastes with higher PA content, such as GCZ1-W, the pozzolanic reaction contributes more significantly to strength development. It is likely that the pozzolanic reaction is the reason why, at the age of 90 days, both zeolite-containing pastes cured under water (GCZ1-W and GCZ0.6-W) exhibit higher strengths than their counterparts cured under air (GCZ1 and GCZ0.6). This assumption is also supported by the XRD and DTA/TG analyses (Section 4.4 and Section 4.5).

Regardless of age, the pastes containing BP as PA and cured under water (GCBP1-W and GCBP0.6-W) consistently show lower compressive strengths than the corresponding air-cured pastes (GCBP1 and GCBP0.6). This might be due to the lower pozzolanic activity of BP, which results in insufficient additional C-S-H formation. Nevertheless, a pozzolanic reaction occurs, since the paste with higher BP content (GCBP1-W) exhibits relatively higher 90-day strength (by about 25% higher) than the paste containing less PA (GCBP0.6-W).

### 4.3. Water Resistance

**Figure 10:**
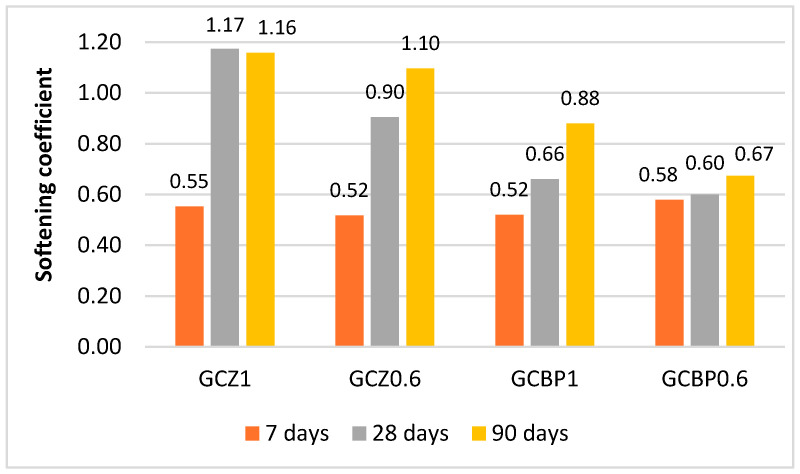
Softening coefficient of pastes at different ages.

The softening coefficient of the investigated GCPB on the 7th day is in the range of 0.52–0.58, indicating that during the early hardening period, these binders are not water-resistant due to the dominance of the hydration processes of gypsum, which is characterized by low water resistance [14,51]. After 28 days, the softening coefficient of the zeolite-containing pastes (GCZ1 and GCZ0.6) exceeds 0.8, which classifies them as water-resistant (Figure 10). Values above 1.0 for the zeolite-containing pastes indicate more effective structural reactions (hydration and pozzolanic reaction) under water than under air conditions.

For the pastes with lower brick powder content (GCBP0.6), the softening coefficient remains below 0.8 even at the age of 90 days, making them unsuitable for wet environments or submersion. In contrast, the paste with higher BP content (GCBP1.0) reaches a coefficient of 0.88 after three months, formally satisfying the water-resistance criterion.

### 4.4. XRD

The XRD analysis of the GCPBs shows that the zeolite-containing pastes (GCZ1 and GCZ0.6) have a slightly higher X-ray amorphous phase (around 26% after 90 days of water curing and 22% after air curing) compared to the BP-containing pastes (GCBP1 and GCBP0.6), where the corresponding values are 25% and 18%. The amorphous phase is mainly due to the unhydrated cement [98], the amorphous components of Z and BP, as well as to the X-ray amorphous nature of most calcium silicate hydrates.

**Figure 11:**
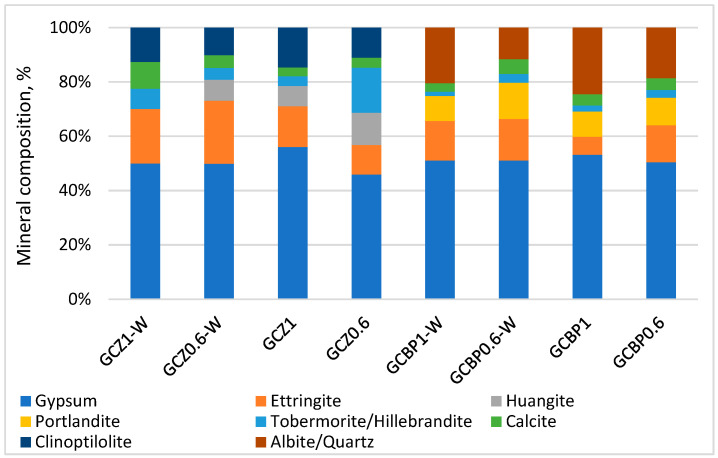
Mineral composition of the crystalline phase at the age of 7 days.

**Figure 12:**
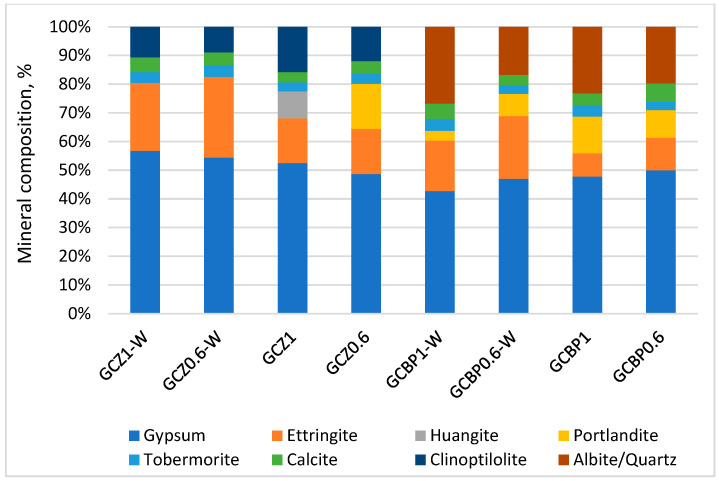
Mineral composition of the crystalline phase at the age of 28 days.

**Figure 13:**
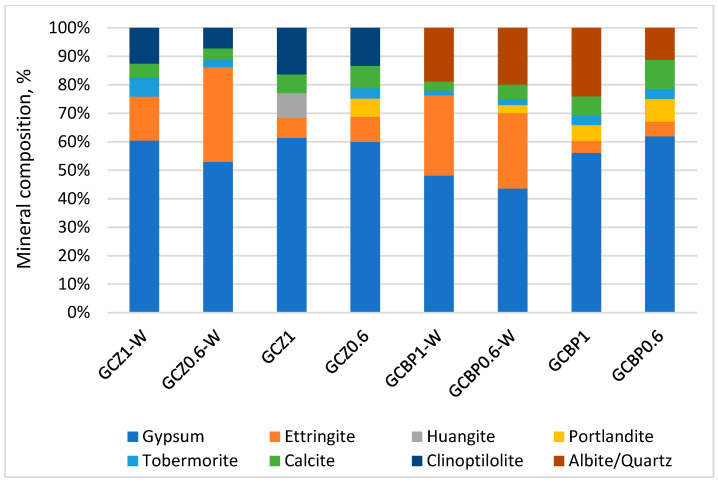
Mineral composition of the crystalline phase at the age of 90 days.

In the 7-day-old pastes, calcium sulfate dihydrate constitutes the largest amount of the GCPB crystalline components. It is formed from the hydration of the recycled gypsum (Figure 11). The content of calcium sulfate dihydrate changes from day 7 to day 90, depending on the curing conditions and the type of PA (Figure 11, Figure 12 and Figure 13).

By day 90, in the water-cured zeolite-containing pastes (GCZ1-W and GCZ0.6-W), the amount of calcium sulfate dihydrate had increased as a result of the ongoing gypsum hydration [61], whereas in the pastes containing BP (GCBP1-W and GCBP0.6-W), the dihydrate content decreased. This is most likely due to the lower pozzolanic activity of BP, which limits the formation of sufficient C-S-H to encapsulate and protect the gypsum crystals from partial dissolution in water [15,56]. As already established, the BP-containing pastes also exhibit lower water resistance (see Section 4.3).

The absence of a pronounced pozzolanic reaction of portlandite with BP in the water-curing GCBP1-W and GCBP0.6-W pastes leads to an increase in the amount of ettringite—on the 90th day, it reaches about 27%. As expected, the air curing does not favor ettringite formation: in GCBP1 and GCBP0.6 pastes the amount of ettringite at the age of three months is only about 5%.

The amount of ettringite is also higher in the water-cured zeolite-containing pastes, (GCZ1-W and GCZ0.6-W). The PA content in the paste plays a significant role: on the 90th day, in the paste with less zeolite (GCZ0.6-W), ettringite accounts for about 30%, whereas in the paste with higher zeolite content (GCZ1-W), it is about 15%. This difference is likely due to a more intensive pozzolanic reaction in the presence of a larger amount of zeolite. This assumption has been supported by the increased C-S-H content (about 7% in GCZ1-W and about 3% in GCZ0.6-W), as well as by the absence of portlandite in the diffractograms. In general, in the zeolite-containing pastes, portlandite is observed only in the air-cured paste containing less zeolite (GCZ0.6). However, on the 90th day its content is twice lower than on the 28th day.

In all pastes, though, small amounts of portlandite are likely present, but due to the sample preparation for XRD analysis, this portlandite carbonates to calcite. The calcite content is approximately 4% in GCZ0.6-W and GCZ1.0-W, and around 6% in GCZ0.6 and GCZ1.0.

In the BP-containing pastes, 3-month-old portlandite is absent only in the air-cured paste with higher BP content (GCBP1-W), while in the other pastes it ranges from about 3% in GCBP0.6-W to 8% in GCBP0.6. The calcite content is also higher in the pastes with BP than in those with zeolite.

**Figure 14:**
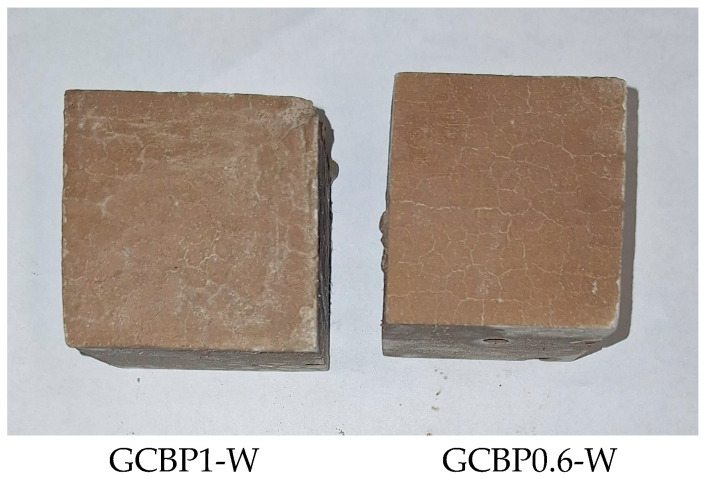
Surface view of 90-day-old water-cured GCPB pastes containing brick powder pozzolanic additive (samples of ca. 40 × 40 × 40 mm, obtained by cutting samples 40 × 40 × 160 mm).

There is a good correlation between the strength development over time and the XRD results—in the pastes containing the insufficiently active PA from brick powder (GCBP1-W and GCBP0.6), ettringite continues to form, especially under water curing, which might be the reason for the lower strengths of these pastes on the 28th and 90th days compared to those containing zeolite, as well as for the low softening coefficient (Section 4.3). The destructive effect of ettringite in these pastes is responsible for the appearance of visible cracks on the surface of the specimens (Figure 14).

**Figure 15:**
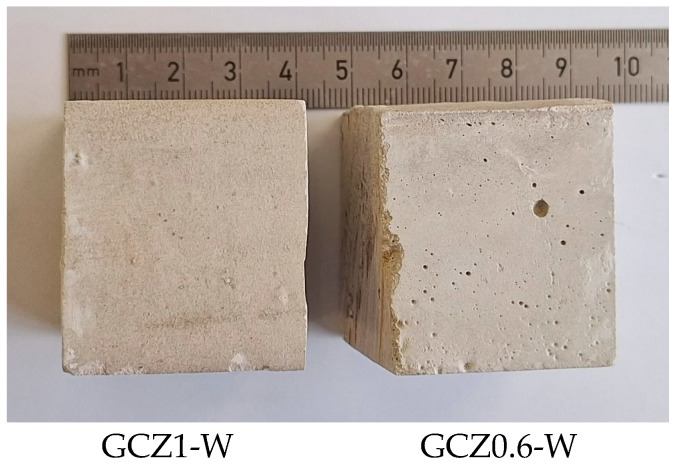
Surface view of 90-day-old water-cured GCPB pastes containing zeolite pozzolanic additive (samples of ca. 40 × 40 × 40 mm, obtained by cutting samples 40 × 40 × 160 mm).

For the corresponding zeolite-containing pastes, no such cracks are observed (Figure 15).

### 4.5. DTA/TG

**Figure 16:**
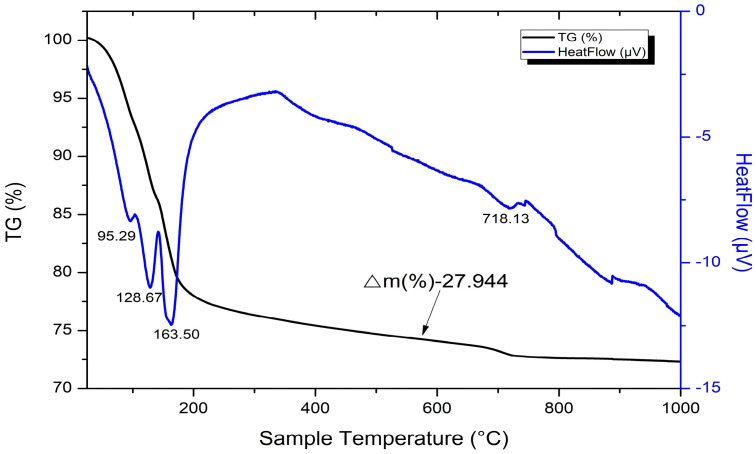
Results of the DTA/TG analysis for the 28-day-old water-cured zeolite-containing GCZ0.6-W paste.

**Figure 17:**
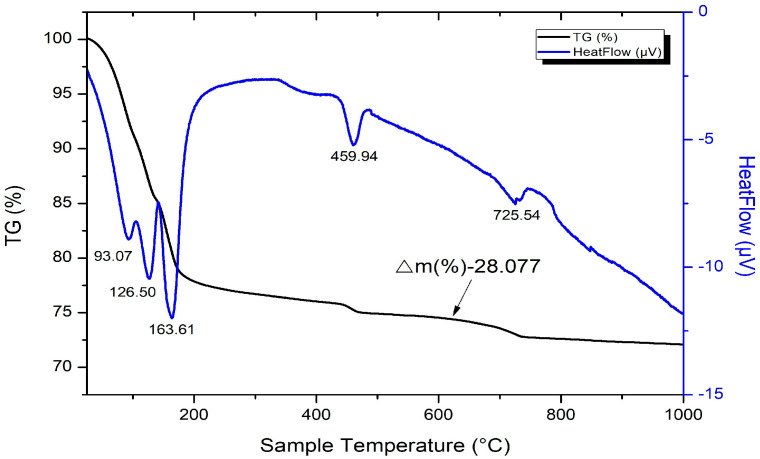
Results of the DTA/TG analysis for the 28-day-old water-cured BP-containing GCBP0.6-W paste.

The DTA/TG analysis confirms the main trends in the structure formation of the investigated ternary GCPB systems, determined by the XRD analysis. Five main peaks can be distinguished, characterizing the following processes: in the temperature range of 30 °C to 110 °C, the release of physically bound water occurs; the peak around 120 °C corresponds to the dehydration of ettringite; the dehydration of gypsum dihydrate and its transformation to bassanite occurs around 160 °C; while the decomposition of portlandite and calcite occurs at approximately 460 °C and 730 °C, respectively (Figure 16 and Figure 17).

An overlap is observed in the temperature range between 140 °C and 340 °C, where several processes co-occur—partial dehydration of C-S-H and the transformation of bassanite into anhydrite. The main difference between the various DTA/TG curves is that the peak characteristic of portlandite decomposition is clearly expressed in some pastes, such as GCBP0.6-W (Figure 17), while in others, such as GCZ0.6-W, it is absent (Figure 16).

**Figure 18:**
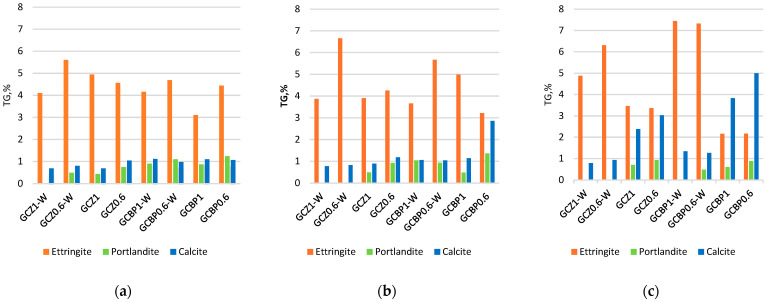
The contents of hydration products in the specimens calculated by TG results: (**a**) pastes at the age of 7 days; (**b**) pastes at the age of 28 days; (**c**) pastes at the age of 90 days.

Analogous to the findings from the XRD analysis, the pozzolanic reaction is more intensive when zeolite is applied, especially when the specimens are under water curing—the amount of portlandite in these pastes is lower than in those containing brick powder, or even entirely absent (Figure 18). Prolonged water curing (up to 90 days) favors ettringite formation (Figure 18c), regardless of the type or content of the pozzolanic additive.

The specific type of calcium hydro sulfoaluminate hydrate cannot be identified, since the peaks of the trisulfate and the lower/monosulfate forms are very close. The amount of calcite in air-cured pastes increases with time, again most likely due to carbonation. According to [61], two long-term competing reactions occur in these ternary systems that consume portlandite—carbonation and the pozzolanic reaction. When carbonation is more intensive than the pozzolanic reaction, the amount of calcite is higher, and vice versa. Water-cured pastes contain less calcite than the air-cured, which indicates that a greater portion of the portlandite is consumed by the pozzolanic reaction.

## 5. Conclusions

It has been determined that recycled gypsum from waste plasterboard can be used to produce blended binders with a gypsum content of above 40%, despite the fact that recycled gypsum exhibits certain differences compared to conventional construction gypsum: lower bulk density (by up to 20%), increased water demand (by about 30%), faster setting and hardening, and lower compressive strength (by up to 20%).

Plasticizing admixtures can be employed to overcome the poor workability of the blended binders.

The natural zeolite used (of Bulgarian origin), with no additional thermal activation, is a suitable pozzolanic additive for these blended binders based on recycled gypsum. Brick powder from recycled construction waste can also be utilized as a pozzolanic additive, but the GCPBs exhibit lower performance. A pozzolanic additive-to-cement ratio of 1.0 ensures higher mechanical properties and achieves water resistance of the pastes after 28 days.

Curing conditions and the amount of PA also play a crucial role in the performance of these blended binders. Prolonged water curing favors both the pozzolanic reaction and ettringite formation. In the water-cured zeolite-containing pastes, the pozzolanic reaction is more intensive (resulting in higher strengths) than in the air-cured ones. For the binders containing brick powder, the pozzolanic reaction is not intensive enough to form sufficient C-S-H to fully protect the gypsum crystals from dissolution in water. For this reason, BP-containing GCPBs are not water-resistant, and under prolonged water curing, ettringite formation might cause destructive processes.

The developed blended binders are fast-setting. The studied mixes exhibit a wide range of compressive strengths, up to 20 MPa with brick powder and up to 30 MPa with zeolite, providing opportunities for optimization according to the intended application.

The developed blended binders can be used in the production of mortars, masonry units, and lightweight composites, and some feasibility and durability studies are already ongoing. The moisture-related environmental conditions can be specified as follows: (a) Blended binders based on recycled gypsum with a zeolite pozzolanic additive can be employed in high-humidity environments without restrictions; (b) When recycled brick powder is used as the pozzolanic additive, however, it is advisable to apply these binders in environments with normal humidity only.

For practical deployment, the initial study on the economic viability of the CDW gypsum recycling in Bulgaria [99] will be further expanded by investigations on the construction products. The most suitable applications of the investigated blended binders will be determined based on the life cycle assessment (LCA) of the products containing them, with the aim of minimizing the environmental footprint.

## Figures and Tables

**Table 1:** Physicomechanical properties of recycled gypsum (RG), compared to conventional construction gypsum (CCG).

Properties	Units	Methods	Recycled Gypsum (RG)	Binder Type “A” (CCG)
Bulk density	kg/m^3^	EN 459-2 [77]	720	875
Water/plaster ratio	-	EN 13279-2 (4.3.1) [78]	0.99	0.66
Initial setting time	min	EN 13279-2 (4.4.1) [78]	3′25″	6′38″
Final setting time	min	prEN 13279-2 [79]	7′36″	13′51″
Flexural strength	N/mm^2^	EN 13279-2 [78]	2.5	3.3
Compressive strength			11.8	14.2
Density of hardened paste	kg/m^3^	EN 12390-7 [80]	863	1105

**Table 2:** Physicomechanical properties of the Portland cement used.

Properties		Units	Methods	Values
Fineness		cm^2^/g	EN 196-6 [82]	3930
Flexural strength	2 days	N/mm^2^	EN 196-1 [83]	6.5
	28 days			9.8
Compressive strength	2 days			31.5
	28 days			53.9

**Table 3:** Pozzolanic activity of the additives based on the CaO bound.

Amount of CaO Bound by 1 g Additive After 30 Days [mg]	Additive Activity
below 50	low activity
50–80	medium activity
80–150	high activity

**Table 4:** XRD of the zeolite PA used.

Amorphous Phase	Clinoptilolite-Na	Heulandite-Ca	Cristobalite-Low
23.0	43.5	55.3	1.3

**Table 5:** XRD of the brick powder.

Amorphous Phase	Quartz	Мuskovite	Calcite	Albite	Microcline	Hematite
27.5	50.8	9.1	2.0	15.5	21.6	1.0

**Table 6:** Composition of gypsum-cement-pozzolan binders.

Mix Designation	RG, % Per Total Binder	OPC, % Per Total Binder	PA, % Per Total Binder	Water/Binder	Admixture, % Per Mass of OPC and RG
GCZ1	45.0	27.50	27.50	0.7	1.2
GCZ0.6	45.0	34.37	20.63	0.7	0.8
GCBP1	45.0	27.50	27.50	0.7	0.8
GCBP0.6	45.0	34.37	20.63	0.7	0.4

## Data Availability

The original contributions presented in this study are included in the article. Further inquiries can be directed to the corresponding author.

## Abbreviations

The following abbreviations are used in this manuscript:

BP	Brick powder
CaO	Calcium oxide
CCG	Conventional construction gypsum
CDW	Construction and demolition waste
CH	Calcium hydroxide
C-S-H	Calcium Silicate Hydrates
HCl	Hydrochloric acid
EN	European Norm
DTA/TG	Combined Differential Thermal Analysis and Thermogravimetry
G	Gypsum
GCPB	Gypsum-cement-pozzolan binder(s)
OPC	Ordinary Portland cement
PA	Pozzolanic additive(s)
RG	Recycled gypsum
XRD	X-ray diffraction
Z	Natural zeolite
